# Supplementary material for: Efficacy of Plant-Made Human Recombinant ACE2 against COVID-19 in a Golden Syrian Hamster Model
Source: Viruses. 2023 Apr 14;15(4):964. doi: 10.3390/v15040964 (PMC10146983; doi:10.3390/v15040964)
Supplement: Supplementary file 1 [file viruses-15-00964-s001.zip › ACE2-Supplementary_Figure_S1.pdf]

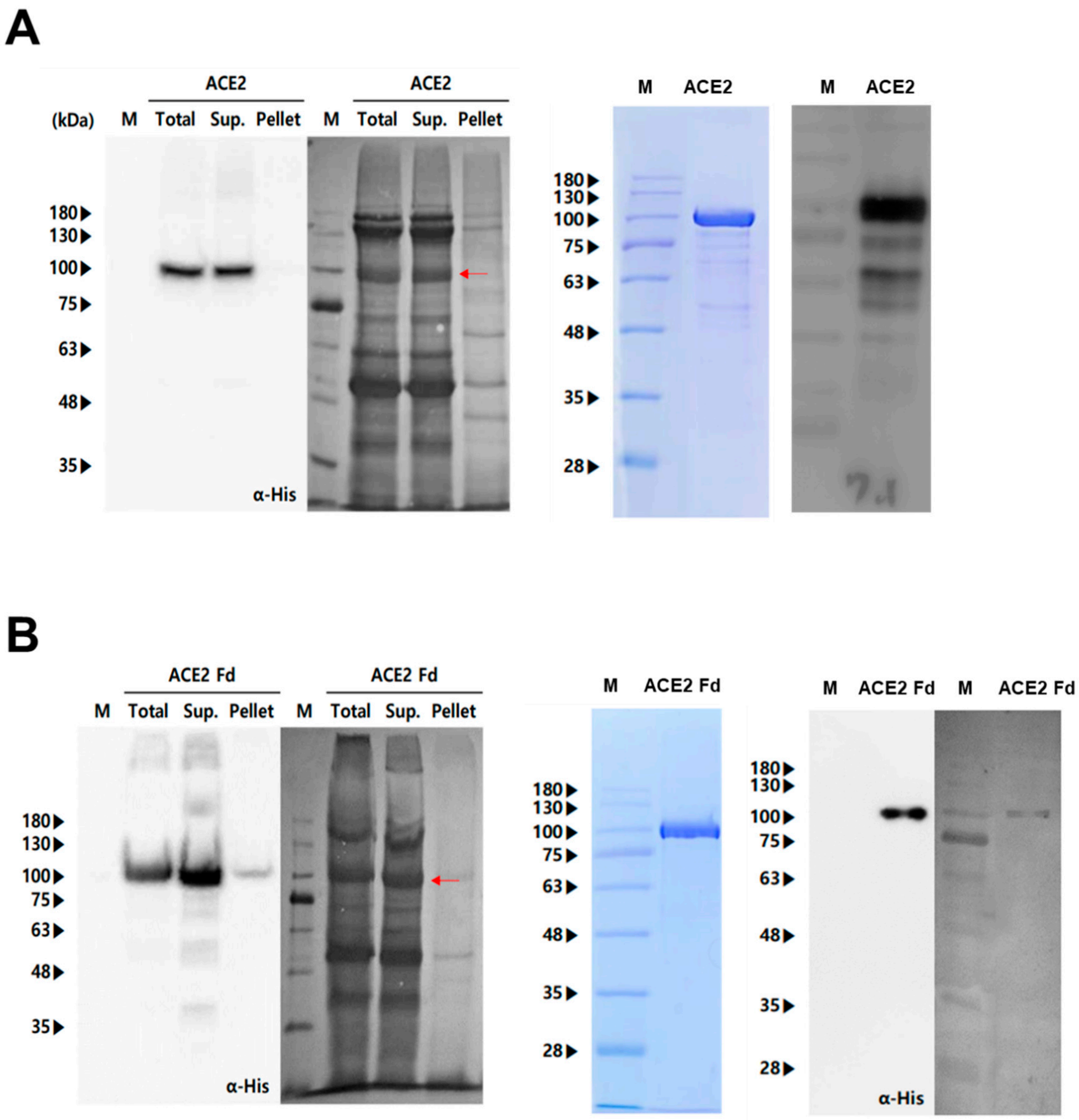

Supplementary Figure S1. SDS-PAGE and western blot analysis of purified hrACE2 (A) and hrACE2-Fd (B) recombinant proteins. hrACE2 and hrACE2-Fd expression in the total protein extract and in soluble and insoluble fractions was assessed using western blotting with the anti-His antibody (α-His). Confirmation of the purified hrACE2 and hrACE2-Fd was done by SDS-PAGE stained with Coomassie blue and western blotting with the anti-His and rabbit anti-ACE2 polyclonal antibodies (Sino Biological, #80031-RP02).
