# Supplementary material for: Efficacy of Plant-Made Human Recombinant ACE2 against COVID-19 in a Golden Syrian Hamster Model
Source: Viruses. 2023 Apr 14;15(4):964. doi: 10.3390/v15040964 (PMC10146983; doi:10.3390/v15040964)
Supplement: Supplementary file 1 [file viruses-15-00964-s001.zip › ACE2-Supplementary_Table_S1.pdf]

Supplementary Table S1. Modified Ashcroft scale (lung fibrosis) of lung tissue lesions among hamster groups at different time points[*].

Groups	dpi[*]	Case no.	Grade of fibrosis[¶]
G1 PBS control		1	0
		2	0
		3	0
		4	0
G2 virus infection	3	1	1
		2	4
	6	1	6
		2	5
	9	1	7
G3 ACE2 Injection	3	1	2
		2	2
		3	1
	6	1	5
		2	5
		3	5
	9	1	2
		2	3
G4 ACE2-Fd Injection	3	1	5
	6	1	6
		2	5
	9	1	3
		2	3
		3	5

[*] Dpi, days post infection;

[¶] 0, Normal with no fibrosis; 1, isolated thick septum; 2, isolated knot-like formation; 3, contiguous fibrotic walls, no fibrotic mass; 4, single fibrotic mass (≤10% of microscopic field); 5, confluent fibrotic mass (>10% and ≤50% of microscopic field); 6, large contiguous fibrotic mass (>50% of microscopic field); 7, alveoli nearly obliterated with honeycombing.
